# Aging Studies on Food Packaging Films Containing β-Cyclodextrin-Grafted TiO_2_ Nanoparticles

**DOI:** 10.3390/ijms22052257

**Published:** 2021-02-24

**Authors:** Leire Goñi-Ciaurriz, Marta Senosiain-Nicolay, Itziar Vélaz

**Affiliations:** Department of Chemistry, Faculty of Sciences, University of Navarra, 31080 Pamplona, Spain; lgoni.4@unav.es (L.G.-C.); msenosiain.4@alumni.unav.es (M.S.-N.)

**Keywords:** TiO_2_ nanoparticles, β-Cyclodextrin, polyvinyl alcohol, ethylene–polyvinyl alcohol copolymers, film degradability

## Abstract

Polymeric materials, such as polyvinyl alcohol (PVA) and ethylene–PVA copolymers (EVOH) are widely used in the food sector as packaging materials because of their excellent properties. TiO_2_ nanoparticles (NPs) show photocatalytic activity; when added to the aforementioned polymers, on the one hand, they are expected to provide bactericidal capacity, whereas on the other hand, they could favor nanocomposite degradation. These types of nanoparticles can be derivatized with cyclodextrin macromolecules (CDs), which can act as food preservative carriers, increasing the packaging food protective properties. In this work, films containing β-Cyclodextrin (βCD)-grafted TiO_2_ nanoparticles and PVA or EVOH were prepared. Regarding the photocatalytic activity of the nanoparticles and the possible environmental protection, accelerated aging tests for PVA, EVOH, and their composites with cyclodextrin-grafted TiO_2_ nanoparticle (NP) films were performed by two methods, namely, stability chamber experiments at different conditions of temperature and relative humidity and UV light irradiation at different intensities. After analyzing the systems color changes (CIELAB) and Fourier transform infrared spectroscopy (FTIR) spectra, it was observed that the film degradation became more evident when increasing the temperature (25–80 °C) and relative humidity percentage (28–80%). There was no significant influence of the presence of CDs during the degradation process. When irradiating the films with UV light, the largest color variation was observed in the nanocomposite films, as expected. Moreover, the color change was more relevant with increasing NP percentages (1–5%) due to the high photocatalytic activity of TiO_2_. In addition, films were characterized by FTIR spectroscopy and variation in the signal intensities was observed, suggesting the increase of the material degradation in the presence of TiO_2_ NPs.

## 1. Introduction

In the food industry, regarding the economic and environmental costs involved, many aspirations focus on improving food preservation in certain ways, including the packaging of perishable products. The wide use of thermoplastic polymers as food packaging materials is attributed to their barrier properties and their fluidity, moldability, and heat sealability under production processes, allowing food protection, storage, and distribution [1]. A particular case is the use of polymeric materials in the so-called “active packaging”; the material itself interacts with the food and improves safety or sensory properties while maintaining nutritional quality, for example, by releasing specific molecules to delay the expiration date [2,3]. Some examples of active packaging include oxygen and ethylene scavengers and antimicrobial and antioxidant films [4,5,6,7].

Active packaging incorporates an active agent or chemical element, which is key in the preservation of packaged food. In general, two approaches are used: (a) introduction of the active substance together with the product in a small bag or envelope, or (b) incorporation of the active element into the packaging as an additive. This second option is more attractive to the consumer, because it avoids incorporating foreign elements into the food itself.

Polymeric materials, such as polyvinyl alcohol (PVA) and ethylene–PVA copolymers (EVOH) are widely used in the food industry as packaging materials because of their excellent properties. PVA shows benefits such as water solubility, high chemical and thermal resistance, high elasticity, and excellent film-forming properties [8,9], and EVOH gained recognition because of its barrier properties against gases due to the strong intermolecular and intramolecular bonding produced by the polar hydroxyl groups present in the vinyl alcohol unit [10,11]. However, the use of these polymers is limited because of their challenging disposal. EVOH is a relatively low degradable polymer, commonly blended with other compounds to increase the decomposition rate [12]. PVA, however, is a biodegradable polymer but it is difficult to biologically degrade it without suitably adapted bacteria strains [13,14].

Titanium dioxide (TiO_2_) is a common food additive recognized as safe (GRAS) by the United States Food and Drug Administration (FDA) [15]. When TiO_2_ is irradiated with light of wavelength lower than 387 nm, it generates enough energy to induce the formation of reactive oxygen species (ROS), such as hydroxyl radicals (OH∙) and superoxide radicals (O_2_^−^) [16,17]. These species were demonstrated to degrade cell components from a wide range of microorganisms and act as antibacterial agents through a redox mechanism [18,19]. TiO_2_ is also a widely used nanomaterial applied in packaging systems due to its high photocatalytic performance that acts as an oxygen and water scavenger and preservative [20]. Some paired electron (e^−^)-holes (h^+^) produced in the TiO_2_ network activated by UV light interact with oxygen and water molecules, which migrate from the package headspace through the plastic pores, generating reactive oxygen species [21].

It was observed that the presence of TiO_2_ NPs in a polymer matrix provides antimicrobial activity against Gram-positive and Gram-negative bacteria, yeasts, and molds [22,23]; in addition, NPs could favor self-polymer degradation under light excitation, using a renewable energy such as the sun [24,25]. Also, a recent study on the evaluation of the migration of TiO_2_ from chitosan films into food revealed that most of the titanium remained in the polymer matrix after migration tests. Only a negligible amount of titanium migrated into the food (<5.44 × 10^−4^% of the total titanium in the chitosan matrix). Indeed, the potential risk of TiO_2_ migration can be excluded [26].

Furthermore, the incorporation of TiO_2_ nanoparticles (NPs) into different polymer-based packaging materials was reported to enhance mechanical properties of the developed films, such as heat resistance and tensile strength, and to reduce permeability [27,28,29]. Additionally, the partially hydroxylated TiO_2_ NP surface facilitates the reaction with other active molecules to increase functionality. Reaching the desired level of functionality sometimes leads to addition of multiple fillers in the same matrix, alongside the implicit limitations of physicochemical compatibility that this involves. The synthesis of multifunctional particles by modification of the NP surface can be a solution by incorporating active molecules that can play different roles (e.g., cyclodextrins) [30]. Natural cyclodextrins (CDs) are cyclic oligosaccharides composed of several units of D-glucopyranose: αCD (six units), βCD (seven units), and γCD (eight units). Their most important feature is the ability to form inclusion complexes with a variety of guest molecules due to a wreath-shaped, truncated cone structure with a slightly apolar cavity and a hydrophilic external surface [31,32]. The masking of undesired tastes and odors, the prevention of microbiological contamination, and the protection of active ingredients against oxidation, light-induced reactions, or heat are some of the applications of CDs in food and packaging [33,34,35]. Furthermore, by incorporating CDs or their complexes into packaging material, some weak points (easily degradable) appear in the structure [33]. The addition of CDs as biodegradable compounds into nonbiodegradable polymer matrices may make them more sensitive to aging processes, such as UV light and heat treatments. In addition, CD hydroxyl groups are susceptible to chemical transformation, making them versatile and improving some properties of native CDs, such as their solubility, controlled delivery of active molecules, and affinity for some guests [36]. The addition of CD to the TiO_2_ NP surface, using a linker as hexamethylene diisocyanate (HMDI), produces a useful nanocarrier system. HMDI is one of the isocyanates approved for food-contact applications [15]. Even though isocyanates are known to be quite toxic, they are used in very low amounts (<0.5%) and all unbonded HMDI chains are removed during the modification process. This system can be incorporated into thermoplastic polymers to obtain films with applications in food packaging. Also, food preservatives can be loaded in the CD cavity. This fact can provide protection against loss and heat decomposition during preparation of the packages, and is also intended to reinforce the TiO_2_ intrinsic antibacterial activity. A previous work reported the loading capacity and further release of different food preservatives from the CD-grafted TiO_2_ NPs [37]. The controlled release of these active molecules from the cavity of the macrocycles may extend the inhibitory effect of the NPs on microbial growth [38].

In this work, films with the βCD-derivatized TiO_2_ NPs dispersed in PVA and EVOH as polymeric matrices were prepared. The purpose of this research was to study the influence of derivatized and commercial TiO_2_ NPs in the aging process of EVOH and PVA. The aim was to know if the additive facilitates the degradation process and, consequently, the disposal of the plastic wastes while ensuring the commercial lifetime of the food packaging polymer. Accelerated aging tests were performed on EVOH, PVA, and their TiO_2_ and βCD-TiO_2_ nanocomposites to study their photo-oxidative and thermo-oxidative behaviors at high temperatures, in a humid atmosphere, and under UV light irradiation. Two methods were employed, namely, stability chamber experiments at different conditions of temperature and relative humidity, and UV light irradiation at different intensities. The EVOH and PVA composites were characterized using Fourier transform infrared spectroscopy (FTIR) and thermogravimetric analysis (TGA).

## 2. Results

### 2.1. Characterization of NP Surface Modification

**Figure 1:**
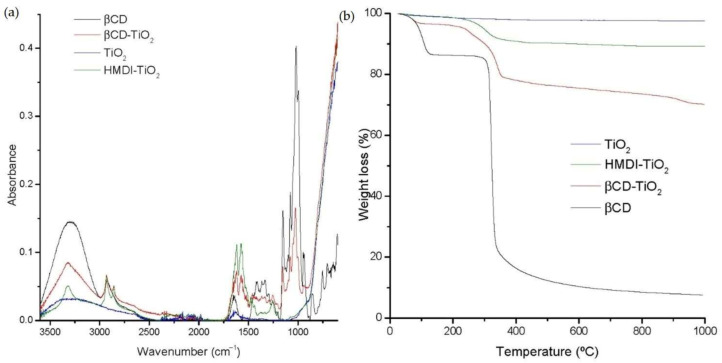
(**a**) FTIR spectra of TiO_2_, HMDI-TiO_2_, βCD and βCD-TiO_2_ NPs. (**b**) TGA curves of TiO_2_, HMDI-TiO_2_, βCD and βCD-TiO_2_ NPs.

In order to characterize the obtained system and to confirm that synthesis occurred correctly, the FTIR spectra corresponding to βCD, modified, and unmodified TiO_2_ NPs, respectively, were constructed, as seen in Figure 1a. Some characteristic bands corresponding to all the reactants were noticed in the spectra of modified NPs, as well as other new functional groups created during the reaction, proving the actual bonding of the βCD to the TiO_2_. For instance, the βCD spectrum showed a characteristic broad band between 900 and 1200 cm^−1^ due to the overlapping of many vibrational modes of C–O–C of glucose and C–OH bonds. The presence of this region in the modified TiO_2_ NPs spectrum confirmed the βCD modification step. Regarding the characteristic vibrations of the linear spacer covalently attached to TiO_2_ NPs and βCD, the hexamethylene diisocyanate (HMDI), βCD-TiO_2_ NP spectrum exhibited a band at ca. 1650 cm^−1^ due to the –C=O groups. Also, in this spectrum, new functional groups originating in the reaction at ≈3300 cm^−1^ and 1600 cm^−1^ were observed, corresponding to N–H stretching and N–H bending modes, respectively. The carbamate groups were formed as a result of the linkage of the spacer to the TiO_2_ and the βCD.

Surface modification of the TiO_2_ NPs was also characterized by TGA. Figure 1b shows the TGA thermograms of βCD, commercial TiO_2_ NPs, TiO_2_-HMDI (first modification), and βCD-TiO_2_ NPs (second modification). TiO_2_ NPs presented a flat profile up to 1000 °C. In the case of the βCD curve, two weight losses were noted, below 100 °C and above 320 °C, lasting up to 1000 °C. The first one was attributed to water loss of the sample, and the second one corresponded to the βCD thermal decomposition. The HMDI-NPs sample displayed a main weight loss starting at around 240 °C assigned to the decomposition of the HMDI chains linked to the TiO_2_ NPs. Lastly, the βCD-TiO_2_ NPs curve exhibited a higher drop in a two-step thermal decomposition process attributed to the removal and decomposition of HMDI and βCD from the nanosystem up to 1000 °C, respectively. The amount of CDs present in the batch was obtained through the difference in the percentage of weight loss between HMDI-NPs and βCD-NPs. Thereby, the βCD-grafted NPs contained 17% βCD. 

### 2.2. Accelerated Aging Tests of the Polymeric Films. Evaluation of Degradation

Films of PVA and EVOH containing 0, 0.1, 0.5, 1, 2, and 5 wt% of TiO_2_ NPs and 1 and 5% βCD-TiO_2_ NPs were prepared and accelerated. Aging assays under different conditions of UV radiation and temperature and relative humidity were carried out. The degradation process was monitored by the study of the color changes and the FTIR spectra obtained.

#### 2.2.1. Degradation Studies in Temperature and Humidity Stability Chamber

Accelerated experiments were performed on EVOH and PVA composites to evaluate the pro-oxidant action of TiO_2_ and βCD-TiO_2_ in the absence of UV light. Color changes, L* (lightness), a* (red–green color), and b* (yellow–blue) parameters were recorded to analyze the degradation of the films after exposure to different conditions in the temperature humidity stability chamber. The L*, a*, and b* values of a nonprocessed sample using the same composition were taken as a reference to compare all other samples. In addition, the total color difference parameter (∆E) was obtained. A threshold value of ∆E = 1 was assumed to be a perceptible color change by human eye [39]. A color change of thermoplastic polymers typically entails intensive thermo-oxidative or photo-oxidative degradation [40,41,42]. Table 1 and Table 2 report the average values of color parameters L*, a*, b* (measured in triplicate), and the total color variation (∆E) measured according to the CIELAB system of samples of the EVOH and PVA composites, respectively, to visually evaluate the overall processes taking place. Samples were processed by heating (25 to 80 °C) and moistening (fixed 80% relative humidity) for time intervals ranging from one to seven weeks (first week and after every two weeks). For comparison purposes, Table 1 and Table 2 display only the results corresponding to the nanocomposites containing 1 and 5 wt% NPs and derivatized NPs (data from 0.1, 0.5, and 2 wt% NPs are omitted).

Table 1 shows the chromaticity values of the EVOH samples as a function of aging at different temperatures. The luminosity (L*) of the EVOH films remains practically unchanged while varying the composition and experimental environmental conditions in the climate chamber, except for the 5% TiO_2_ NPs composite, which underwent a slight decrease after 80 °C temperature and 80% humidity processing. The changes in the chromaticity value of a* on the studied EVOH composites were negligible throughout the aging tests. The most significant color variation on the polymer composites as the temperature rises was the yellowing of the material. It was indicated by the increase of parameter b* (blue–yellow axis) on the CIELAB scale, which, according to Cai [41] is the most accurate colorimetric coordinate to evaluate the potential degradation of polymers. Moreover, the yellowing of the material became more noticeable when increasing the NP concentration. The highest b* values were obtained with 5% TiO_2_ and 5% βCD-TiO_2_. The presence of βCD in the NPs does not significantly influence this parameter.

The results showed remarkable differences in the total color variation (∆E > 5), which enabled a quantitative comparison of the color changes of the polymers. These changes were caused by polymer thermo-oxidative and photo-oxidative degradation [43]. In the case of the EVOH polymer, the 5% TiO_2_ and 5% βCD-TiO_2_ composites experienced a substantial change in color at 60 °C, ∆E = 9.7, and ∆E = 5.5, respectively. At the highest temperature (80 °C), composites with 5% additive showed a substantial change in color, i.e., ∆E above 12. The color variations in presence and absence of βCD were minimal, taking into account the reduction in TiO_2_ when βCD was present, which was more remarkable in the case of 1% NPs.

The data obtained for parameters on PVA polymer matrix after processing at various temperatures are shown in Table 2. The luminosity (L*) of the samples declined to a small extent when increasing the temperature and the amount of additive in the polymeric matrix. The variations in L* parameter were similar in all the cases, with the exception of the significant variation observed in 5% βCD-TiO_2_ PVA. Here, L* decreased up to 0.16-fold after processing at the highest temperature (80 °C). Also, in this case, there was no significant impact of the processing parameters on the chromaticity value of a*, except for the 5% βCD-TiO_2_ performed at 80 °C, which changed slightly to a reddish coloration (a* turned to positive value). The chromaticity value of b* increased gradually with the temperature and the amount of additive in the polymer matrix. The PVA matrix showed more yellow with 5% βCD-TiO_2_ content with regard to the 5% TiO_2_ filling, as was the case of the 1% NPs.

Concerning ∆E, a significant color variation of 5.2 was observed in the 5% TiO_2_ composite at a lower temperature of 40 °C. The difference in color ∆E between 5% TiO_2_ and 5% βCD-TiO_2_ was much higher at 80 °C: 22 and 31, respectively. Thus, in these extreme conditions, the temperature and the presence of βCD in the PVA matrix seemed to facilitate the aging of the film.

The second variable parameter of the assay, relative humidity (28–80%) showed no significant color variations (∆E < 5) in time at a fixed temperature (25 °C) for any of the EVOH and PVA composite films.

Considering the data in Table 1 and Table 2, it can be concluded that temperature decreased lightness L*, increased the b* parameter (blue–yellow axis), and barely changed the a* value (green–red axis) in all the EVOH and PVA samples. The TiO_2_ NPs and βCD-grafted TiO_2_ additives acted as influential pro-oxidants under dark and moisten thermo-oxidative conditions. The PVA composites are noted to be more susceptible to weathering conditions than EVOH films; more intense discoloration when processing at high temperatures (up to 80 °C) was therefore observed.

#### 2.2.2. Photodegradation Studies

**Figure 2:**
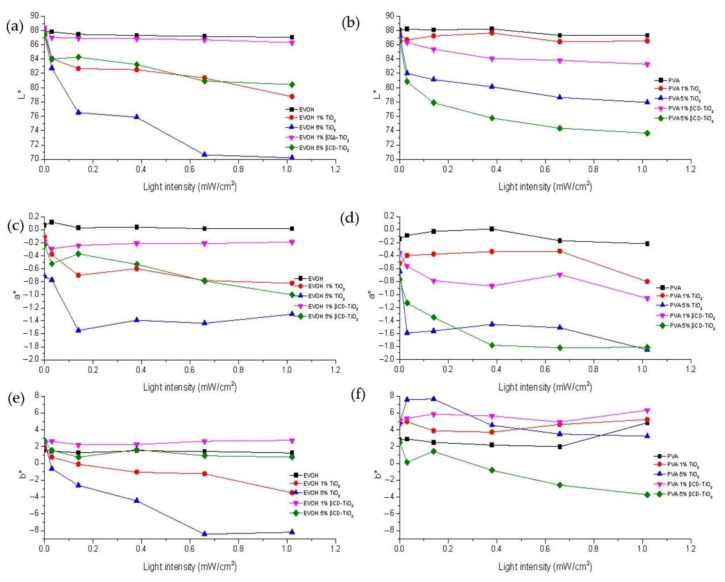
CIELAB values (L*, a*, b*) for EVOH and PVA composite films as a function of UV-A light irradiation intensity. L* coordinates for (**a**) EVOH and (**b**) PVA; a* coordinates for (**c**) EVOH and (**d**) PVA; b* coordinates for (**e**) EVOH and (**f**) PVA.

The three CIELAB coordinates (L*, a*, and b*) for EVOH and PVA composites as a function of aging at different UV light irradiation intensities are exposed in Figure 2.

The luminosity of EVOH and PVA polymers followed a gradual decreasing trend as the light intensity and the amount of additive increased. In the case of the 5% TiO_2_ EVOH composite, the L* value fell from a value of 87.5 when the polymer was unprocessed to a minimum of 70.2 after 24 h under 1.02 mW/cm^2^ UV-A light irradiation. The PVA matrix reached its maximum decrease (L = 73.6) for 5% βCD-TiO_2_ under the most intense irradiation. Regarding the a* parameter, the lowest values were observed for those EVOH and PVA composites with the highest amount of additive (5% TiO_2_ and 5% βCD-TiO_2_ composites), which turned to a greenish tonality under UV light irradiation. The a* coordinate did not show a gradual trend throughout the processing. The composites with the highest amounts of NPs showed a small decline in the b* value, turning into a slightly bluish coloration.

**Figure 3:**
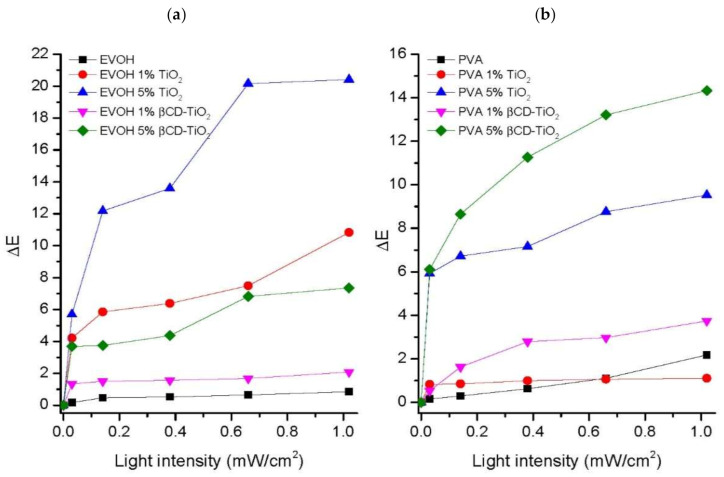
Composite total color variations (∆E) under exposure to different intensities of UV light (365 nm) during 24 h: (**a**) EVOH, EVOH/TiO_2_, and EVOH/βCD-TiO_2_ composites, and (**b**) PVA, PVA/TiO_2_, and PVA/βCD-TiO_2_ composites.

Figure 3 displays the total color variations (∆E) for EVOH and PVA films. UV light is known to have a negligible aging effect on EVOH and PVA polymers [14,24], which supports the low susceptibility to discoloration (∆E < 1) shown by plain EVOH and PVA during up to 24 h of UV light exposure. However, the degradation of the polymeric matrices was shown by the TiO_2_ and βCD-TiO_2_ composites. The color alteration in the case of the EVOH matrix was larger for the TiO_2_ composites than for the βCD-grafted NPs, reaching an ∆E value above 20 (5% TiO_2_). For the PVA polymer, the degradation rates were higher for the βCD-TiO_2_ additive than for TiO_2_ NPs at the same percentage, therefore, βCD-grafted TiO_2_ facilitated PVA degradation under photo-oxidative conditions.

At the end of the experiment, EVOH and PVA plain samples presented the same initial visual aspects, while the TiO_2_ and βCD-TiO_2_ polymer composites showed slightly more colored appearances. This was indicative of the photocatalytic oxidation of polymer matrices by TiO_2_ NPs under UV radiation. The same assay was performed for 48 h of UV light irradiation and similar results were obtained.

#### 2.2.3. FTIR Characterization

**Figure 4:**
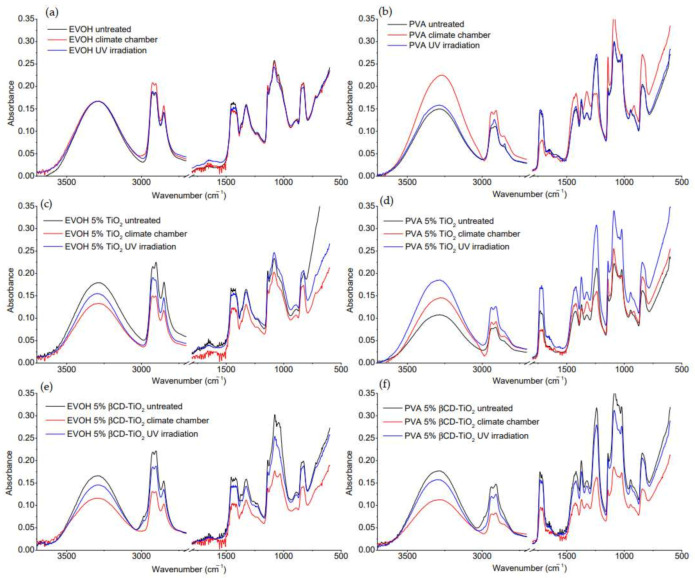
FTIR spectra of films containing (**a**) EVOH, (**c**) EVOH 5% TiO_2_ composites, (**e**) EVOH 5% βCD-TiO_2_ composites, (**b**) PVA, (**d**) PVA 5% TiO_2_ composites, and (**f**) PVA 5% βCD-TiO_2_ composites before and after treatment in a stability chamber (80 °C and 80% relative humidity) and UV light irradiation (365 nm) over 24 h.

The chemical changes of EVOH and PVA films during the treatment in the stability chamber (80 °C and 80% humidity) and UV light irradiation were studied by FTIR spectroscopy. Figure 4 shows the FTIR spectra obtained for the plain polymers and the 5% TiO_2_ and 5% βCD-TiO_2_ composites of EVOH and PVA before and after processing. No additional peaks caused by the applied processing conditions for any of the composites were observed, however, changes in the intensity of the EVOH and PVA characteristic peaks were detected, probably due to polymer degradation, which was more evident than the effect of light irradiation and weathering conditions processing.

The degradation of the EVOH matrix was shown in the case of the 5% TiO_2_ and 5% βCD-TiO_2_-containing nanocomposites by the spectra displayed in Figure 4c,e. The broad band at 3300 cm^−1^ was assigned to EVOH intermolecular and intramolecular hydrogen bonded O–H stretching vibrations. The reduction in intensity of this band after irradiation was noticeable. The transformation of the O–H groups was also detected in the spectra at 1325 and 1437 cm^−1^ by the decrease in the intensity of the peaks associated with O–H deformation modes after processing [25]. The vibration at 1085 cm^−1^ related to C–O stretching hole its absorbance intensity after 24 h of UV light irradiation. These changes suggest that there was a decrease of hydrogen-bonding elements due to irradiation in the presence of TiO_2_, thereby inducing polymer matrix oxidation.

Regarding the PVA spectra, all the characteristic bands can be observed in the spectra (Figure 4b,d,f). There is a broad band around 3300 cm^−1^ corresponding to the stretching vibration of O-H groups from the intermolecular and intramolecular hydrogen bonds. The peaks observed between 2800 and 3000 cm^−1^ referred to the C–H stretching vibrations from the alkyl groups. A strong band at 1090 cm^−1^ was assigned to the stretching vibration of C–O of the C–O–H groups. The band at 1730 cm^−1^ was due to C=O stretching. The intensity of this band was strong for unprocessed and irradiated PVA and 5% TiO_2_ PVA but weak for the climate-chamber-treated films, indicating the loss of a few acetate groups by thermo-degradation [44]. The wide vibrational band between 1200 and 1450 cm^−1^ was the result of the bending and wagging vibrations of CH_2_ groups (1300–1450 cm^−1^) and the C–H wagging (1200–1300 cm^−1^). The processing with strong weathering conditions also resulted in a significant reduction of intensity of the absorption band at 1240 cm^−1^. Photo-oxidation results were also noticeable in the IR spectra. For the plain PVA, the UV light irradiation over 24 h resulted in a 3% and 4% increase in the intensity of the absorption bands at 1730 and 1240 cm^−1^, respectively. In contrast, the photo-oxidation of the 5% TiO_2_ PVA composite led to a significant increase in the intensity of the peaks at 1730 and 1240 cm^−1^, by 48% and 43%, respectively. As no additional bands appeared in the FTIR spectra, it may be concluded that thermo-oxidative and photo-oxidative degradation of EVOH and PVA was not sufficient to create significant amounts of low-molecular-weight polymeric chains. The slight variations between the 5% TiO_2_ EVOH and PVA composite and the 5% βCD-TiO_2_ composite FTIR spectra after processing under the same conditions were attributed to less TiO_2_ present in the grafted NPs. The presence of βCD did not have an influence on the polymer matrix decomposition.

## 3. Materials and Methods

### 3.1. Materials

Titanium (IV) oxide nanoparticles (TiO_2_ NPs 99.5% purity, 21 nm size and ρ = 4.26 g∙cm^−3^) were provided by Sigma-Aldrich (St. Louis, MO, USA). β-Cyclodextrin (βCD 12.5% water content) was manufactured by Roquette (Laisa España S.A.A., Roquette Laisa (Valencia, Spain). Acetone dry (≤0.01% water), *N*,*N*-dimethylformamide dry (DMF), ethanol (96%), ethanol (70%), and methanol (≤0.01% water) were from Panreac Applichem. Hexamethylene diisocyanate (HMDI, 98%) was purchased from Fluka (Morris Planes, NJ, USA). Poly(vinyl alcohol)/Mowiol (PVA) (Mw = 31,000 g/mol) and ethylene–PVA copolymers (EVOH) were procured from Sigma-Aldrich. All reagents were used as received. 

### 3.2. Nanoparticle Surface Modification

The grafting of cyclodextrins (CDs) to the surfaces of TiO_2_ NPs was developed as previously described [30]. In a first modification, a linear spacer, hexamethylene diisocyanate (HMDI) was covalently joined to the hydroxyl groups on the surface of the TiO_2_ NPs by reaction of 4.5 mL of HMDI with 2 g of TiO_2_ NPs in 100 mL of dry DMF medium. The reaction proceeded under nitrogen atmosphere and vigorous magnetic stirring at 100 °C for 72 h. Then, the product was centrifuged at 8000× rpm for 30 min, washed three times with acetone, and left to dry completely at 50 °C in an oven. Afterward, in a second stage, the βCD (previously dried in an oven at 65 °C for 24 h) was covalently bonded to the still free end of the spacer. HMDI was already bonded to the NPs by adding the first step product and 9 g of dry βCD to 100 mL of dry DMF. The reaction proceeded under nitrogen atmosphere and stirring for 24 h at 100 °C. After centrifuging (8000× rpm, 30 min) and washing three times with methanol, the βCD-grafted NPs were left for 24 h at 50 °C in an oven until total dryness to obtain a solid product.

The degree of grafting of CDs to the surface of TiO_2_ was analyzed by thermogravimetric analysis (TGA) and Fourier transform infrared spectroscopy (FTIR). The TGA of the samples was carried out in a TGA-SDTA 851 Mettler Toledo thermobalance. The samples were weighed in alumina crucibles from 25 °C to 1000 °C at 10 °C/min under N_2_ atmosphere. FTIR analysis was performed using an IRAffinity-1S Shimadzu FTIR spectrometer, equipped with a Golden Gate diamond ATR accessory, and spectra were recorded with a resolution of 4 cm^−1^ (32 scans).

### 3.3. Preparation and Characterization of Polymeric Composite Films

EVOH and PVA nanocomposite films were prepared using a solution casting method with different filler content (0, 0.1, 0.5, 1, 2, and 5 wt% TiO_2_ and 1 and 5 wt% βCD-TiO_2_ NPs). Solutions of the selected polymers at 10% *w*/*v* were prepared in a suitable solvent (ethanol for EVOH and water for PVA) by heating under reflux at 90 °C until complete dissolution. Then, TiO_2_ NPs and βCD-grafted NPs at 1 and 5 wt% (by weight of polymer) were dispersed into 10 mL polymeric solution alternating between ultrasonication and vigorous stirring with a magnetic stirrer at room temperature for 1 h. The mixtures were added into individual Petri dishes (10 mm in diameter) and allowed to dry at room temperature for 24 h (prefilms). After that, the material was hot-pressed (Specac, Mini-Film Maker model) at 150 °C and 8 kN for 5 min to acquire the required 100 µm thickness of the films, which were stored in darkness.

Films were characterized by TGA and FTIR using the equipment described in the previous section. The TGA of the samples was performed under a heating rate of 10 °C/min from 25 °C to 1000 °C under N_2_ atmosphere, and FTIR spectra were registered with a resolution of 4 cm^−1^ (32 scans).

### 3.4. Accelerated Aging Tests

Accelerated aging assays were carried out in a VC 0033 Vötsch Industrietechnik-Neurtek chamber under different conditions of temperature and relative humidity. Firstly, the temperature was kept constant while varying the ambient humidity. Then, the assays were performed over seven weeks and the samples were measured every two weeks. The processing parameters and their combinations were collected and are displayed in Table 3.

To evaluate the action of UV light on polymer composites, samples were exposed to UV-A light (λ = 365 nm) at room temperature. The samples were arranged at four distances from the lamp, 6, 12, 18, 24, and 30 cm, and received measured light intensities of 1.02, 0.66, 0.38, 0.14, and 0.03 mW/cm^2^, respectively. The composite films were exposed to UV light irradiation for 24 h and 48 h. Plain films of EVOH and PVA were used as controls.

After aging, the composite samples were characterized and tested by color change according to the CIELAB scale and FTIR analysis.

### 3.5. Evaluation of Composite Films Degradation

#### 3.5.1. Film Color Change

The degradation of the films was assessed by change in color of the samples according to the International Commission on Illumination (CIE) through L*/a*/b* coordinates. In this system, L* is the color lightness (L* = 0 for black and L* = 100 for white), a* indicates chromaticity on a green (−)/red (+) axis, and b* is the blue (−)/yellow (+) axis [45]. The color was determined using a color meter (Model Konica Minolta Spectrophotometer CM-2300d) and the tests were performed in triplicate. The total color difference parameter (∆E) was obtained following Equation (1) [46]:

(1) $$\Delta E={\left[{\left(\Delta L^{*}\right)}^{2}+{\left(\Delta a^{*}\right)}^{2}+{\left(\Delta b^{*}\right)}^{2}\right]}^{1/2}$$

where $\Delta L^{*}=\;L_{sample}^{*}\;-\;L_{standard}^{*};\;\Delta a^{*}=\;a_{sample}^{*}\;-\;a_{standard}^{*};\;\Delta b^{*}=\;b_{sample}^{*}\;-\;b_{standard}^{*}$.

According to EN ISO 2813, the ∆E ranges include: 0 < ∆E < 1: invisible color variation; 1 < ∆E < 2: small variation of color, recognizable only by an experienced observer; 2 < ∆E < 3.5: medium variation of color, recognizable by the inexperienced observer; 3.5 < ∆E < 5: distinct color variation; ∆E > 5: large color variation.

#### 3.5.2. FTIR Film Characterization

The chemical alterations during the aging process were also analyzed by FTIR spectroscopy operated with a resolution of 4 cm^−1^ and 32 scans and covering a domain from 4000 to 600 cm^−1^. The samples were tested by using the attenuated total reflectance (ATR) method (IRAffinity-1S Shimadzu FTIR spectrometer equipped with a Golden Gate diamond ATR accessory).

## 4. Conclusions

In this work, films containing composites of EVOH and PVA and different % TiO_2_− and βCD-grafted NPs were prepared and characterized. Films were treated in a temperature and humidity stability chamber and under different UV light intensities in order to study the influence of TiO_2_ NPs regarding polymer degradation. The system changes were monitored by the color variation of the films (CIELAB) and FTIR characterization. It can be concluded that temperature and UV radiation had a significant impact on the color change of the EVOH and PVA composites studied, but not on the plain matrices. Under the processing conditions, TiO_2_ NPs facilitated the degradation of the polymer matrix due to their photocatalytic activity. The presence of βCD seemed to slightly favor the degradation of the PVA polymeric matrix at the higher temperature studied (80 °C), in contrast to EVOH, which underwent greater oxidation with unmodified TiO_2_ NPs under thermo- and photo-oxidative conditions.

Currently, great efforts are being made to obtain packages for food preservation, which not only prolong food durability but also improve degradability in order to protect the environment. In this sense, the results obtained in this work are promising. On the one hand, the added costs to obtain films containing grafted TiO_2_ NPs are minimal. On the other hand, these systems seem to be stable at temperatures under 40 °C; at higher temperatures, the photocatalytic activity of the nanoparticles favors the degradation of the polymer.

The results obtained in this study provide useful information to achieve progress in the development of thistype of systems, which include the bactericidal activity of the nanoparticles and the ability to carry food preservatives toward better food conservation. Future studies could focus on revealing the synergy between the titanium dioxide antimicrobial activity and the active agent included in the CD.

## Figures and Tables

**Table 1:** L*, a*, b*, and ∆E color parameters obtained for ethylene–PVA copolymers (EVOH) composite films after aging for at different temperatures and 80% relative humidity.

Additive	Temperature							
	25 °C		40 °C		60 °C		80 °C	
	L*/a*/b*	∆E	L*/a*/b*	∆E	L*/a*/b*	∆E	L*/a*/b*	∆E
None	87.4/0.0/2.2	0.7	87.4/0.0/2.2	0.7	87.3/0.0/2.6	1.1	87.2/0.0/2.6	1.2
1% TiO_2_	87.3/−0.1/2.3	0.9	87.6/−0.1/3.0	1.1	85.8/−0.2/3.8	3.0	85.7/−0.3/6.7	5.3
5% TiO_2_	87.7/−0.5/2.8	0.3	88.5/−0.5/2.4	1.0	86.5/−0.9/12	9.7	85.3/−0.8/15	12
1% βCD-TiO_2_	88.6/−0.2/2.5	0.2	87.5/−0.2/3.8	1.5	86.9/−0.2/3.4	1.7	87.1/−0.3/5.8	3.5
5% βCD-TiO_2_	86.8/−0.2/2.1	0.9	86.8/−0.3/3.4	1.0	86.0/−0.6/8.0	5.5	84.1/−0.6/14	12

Ranges of ∆E according to ISO 2813. 0 < ∆E < 1: invisible color variation; 1 < ∆E < 2: small variation of color, recognizable only by an experienced observer; 2 < ∆E < 3.5: medium variation of color, recognizable by the inexperienced observer; 3.5 < ∆E < 5: distinct color variation; ∆E > 5: large color variation.

**Table 2:** L*, a*, b*, and ∆E color parameters obtained for polyvinyl alcohol (PVA) composite films after aging at different temperatures and 80% relative humidity.

Additive	Temperature							
	25 °C		40 °C		60 °C		80 °C	
	L*/a*/b*	∆E	L*/a*/b*	∆E	L*/a*/b*	∆E	L*/a*/b*	∆E
None	88.4/0.0/2.1	0.8	87.5/−0.2/4.0	1.3	85.6/−0.1/3.2	2.6	86.2/−0.2/6.9	4.5
1% TiO_2_	87.1/−0.4/5.2	0.6	88.7/−0.2/2.4	2.7	86.8/0.1/7.9	3.4	86.8/−0.2/8.1	3.4
5% TiO_2_	89.9/−0.6/2.3	3.7	89.3/−0.7/9.7	5.2	78.2/2.1/24	22	83.6/0.5/26	22
1% βCD-TiO_2_	87.4/−0.2/4.8	0.8	88.8/−0.2/2.9	3.0	87.2/−0.6/10	5.0	83.5/−0.9/10	6.0
5% βCD-TiO_2_	87.9/−0.6/2.8	1.5	87.5/−0.7/6.2	3.8	85.7/0.1/20	17	73.8/4.5/31	31

**Table 3:** Processing parameters in the stability chamber.

Assay	Temperature (°C)	Relative Humidity (%)	Time of Exposure (Weeks)
1	25	28	1
2	25	40	2
3	25	60	2
4	25	80	2
5	25	80	1
6	40	80	2
7	60	80	2
8	80	80	2
